# Upregulation of the ferroptosis-related *STEAP3* gene is a specific predictor of poor triple-negative breast cancer patient outcomes

**DOI:** 10.3389/fonc.2023.1032364

**Published:** 2023-03-31

**Authors:** Lifang Yuan, Jiannan Liu, Lei Bao, Huajun Qu, Jinyu Xiang, Ping Sun

**Affiliations:** ^1^ Department of Oncology, Yantai Yuhuangding Hospital, Shandong University, Yantai, China; ^2^ Department of Breast Oncology, Huanxing Cancer Hospital, Beijing, China; ^3^ Department of Oncology, The Affiliated Yantai Yuhuangding Hospital of Qingdao University, Yantai, China; ^4^ Department of Pathology, The Affiliated Yantai Yuhuangding Hospital of Qingdao University, Yantai, China

**Keywords:** triple-negative breast cancer (TNBC), ferroptosis, prognostic signature, overall survival, *STEAP3*

## Abstract

**Objective:**

This study was designed to assess ferroptosis regulator gene (FRG) expression patterns in patients with TNBC based on data derived from The Cancer Genome Atlas (TCGA). Further, it was utilized to establish a TNBC FRG signature, after which the association between this signature and the tumor immune microenvironment (TIME) composition was assessed, and relevant prognostic factors were explored.

**Methods:**

The TCGA database was used to obtain RNA expression datasets and clinical information about 190 TNBC patients, after which a prognostic TNBC-related FRG signature was established using a least absolute shrinkage and selection operator (LASSO) Cox regression approach. These results were validated with separate data from the Gene Expression Omnibus (GEO). The TNBC-specific prognostic gene was identified *via* this method. The STEAP3 was then validated through Western immunoblotting, immunohistochemical staining, and quantitative real‐time polymerase chain reaction (RT-qPCR) analyses of clinical tissue samples and TNBC cell lines. Chemotherapy interactions and predicted drug sensitivity studies were investigated to learn more about the potential clinical relevance of these observations.

**Results:**

These data revealed that 87 FRGs were differentially expressed when comparing TNBC tumors and healthy tissue samples (87/259, 33.59%). Seven of these genes (*CA9, CISD1, STEAP3, HMOX1, DUSP1, TAZ, HBA1*) are significantly related to the overall survival of TNBC patients. Kaplan-Meier analyses and established FRG signatures and nomograms identified *CISD1* and *STEAP3* genes of prognostic relevance. Prognostic Risk Score values were positively correlated with the infiltration of CD4+ T cells (p = 0.001) and myeloid dendritic cells (p =0.004). Further evidence showed that *STEAP3* was strongly and specifically associated with TNBC patient OS (P<0.05). The results above were confirmed by additional examinations of *STEAP3* expression changes in TNBC patient samples and cell lines. High *STEAP3* levels were negatively correlated with half-maximal inhibitory concentration (IC50) values for GSK1904529A (IGF1R inhibitor), AS601245 (JNK inhibitor), XMD8−85 (Erk5 inhibitor), Gefitinib, Sorafenib, and 5-Fluorouracil (P < 0.05) in patients with TNBC based on information derived from the TCGA-TNBC dataset.

**Conclusion:**

In the present study, a novel FRG model was developed and used to forecast the prognosis of TNBC patients accurately. Furthermore, it was discovered that *STEAP3* was highly overexpressed in people with TNBC and associated with overall survival rates, laying the groundwork for the eventually targeted therapy of individuals with this form of cancer.

## Introduction

With an anticipated 290,560 new diagnoses and 43,780 related deaths in the United States in 2022 alone, breast cancer is one of the most common cancers in women. Indeed, breast cancer remains the second deadliest malignancy in women (1), and the current estimated annual incidence and mortality rates for this cancer type in China are 45.29 per 100,000 and 10.50 per 100,000, respectively (2, 3). The prognosis for patients with metastatic breast cancer remains poor (4). Thus, emphasizing the disease’s continued threat to the health of women around the world despite numerous significant improvements in patient detection and treatment.

Different breast cancer subtypes have been identified based on clinical and morphological characteristics, including inflammatory breast cancer, lobular carcinoma, and ductal carcinoma. TNBC is characterized by the absence of HER-2 (Human epidermal growth factor receptor-2), progesterone receptor (PR), or estrogen receptor (ER) expression on tumor cells (5–7). TNBC tumors do not react to endocrine therapy or anti-HER2 antibody therapy because they lack these receptors, and there are no effective treatments for this breast cancer subtype. The poor prognosis of affected individuals is a result of the lack of efficient treatment choices, the highly invasive aspect of this malignancy, its high rates of recurrence, and a high potential for metastasis (8, 9). Of the different molecular subtypes of breast cancer, TNBC accounts for approximately 12% of cases on average (10), yet it accounts for 40% of breast cancer-related mortality (11, 12). The overall prognosis of TNBC is still unsatisfactory despite the use of treatments such as PARP inhibitors, immune checkpoint inhibitors (ICI), and novel antibody-drug conjugates (ADC) in clinical treatment by targeting specific mutations, proteins, and immune cell types (13–15). Clinically, approximately 70% of TNBC patients respond well to the current treatments and their prognosis is as good as that of the Luminal Breast Cancer subtype. Consequently, our efforts must be oriented to understand why the other 30% of TNBC cases do not respond well to current treatments. Therefore, new, efficient treatments for TNBC patients must be developed.

The iron-dependent cell death process known as ferroptosis is unique from necrosis, apoptosis, and autophagy on a molecular, genetic, and morphological level and is accompanied by substantial lipid peroxidation. Ferroptosis is closely related to many human malignancies (5, 16–18), and a growing understanding of the regulation of ferroptosis within tumor cells suggests that ferroptosis induction may represent an effective treatment strategy (19–22). According to a recent study, the traditional GSH/GPX4, FSP1/DHODH/CoQ10, and GCH1/BH4 pathways are not the only ones that affect ferroptosis activity. The metabolite indole-3-pyruvic acid (in3py), which is produced by the amino acid oxidase interleukin-4-induced-1 (IL4i1), was initially identified as a gene induced in B cells in response to IL-4, suppresses ferroptosis through a radical scavenging mechanism and by orchestrating a gene expression profile that attenuates ferroptosis (23). The therapeutic use of knowledge about ferroptosis may depend on the selective activation or inhibition of these genes in certain tissues, cells, and disease situations (24). Jingjing Du et al. also found that Shuganning (SGNI), a Chinese patent medicine, can selectively inhibit the proliferation of TNBC cells *in vitro* and *in vivo* by inducing ferroptosis (9). In addition, some studies have found that certain genes in TNBC cells regulate ferroptosis activity (25, 26). Moreover, the regulation of ferroptosis and the association between ferroptosis genes and prognostic effects in TNBC remains unknown.

**Figure 1:**
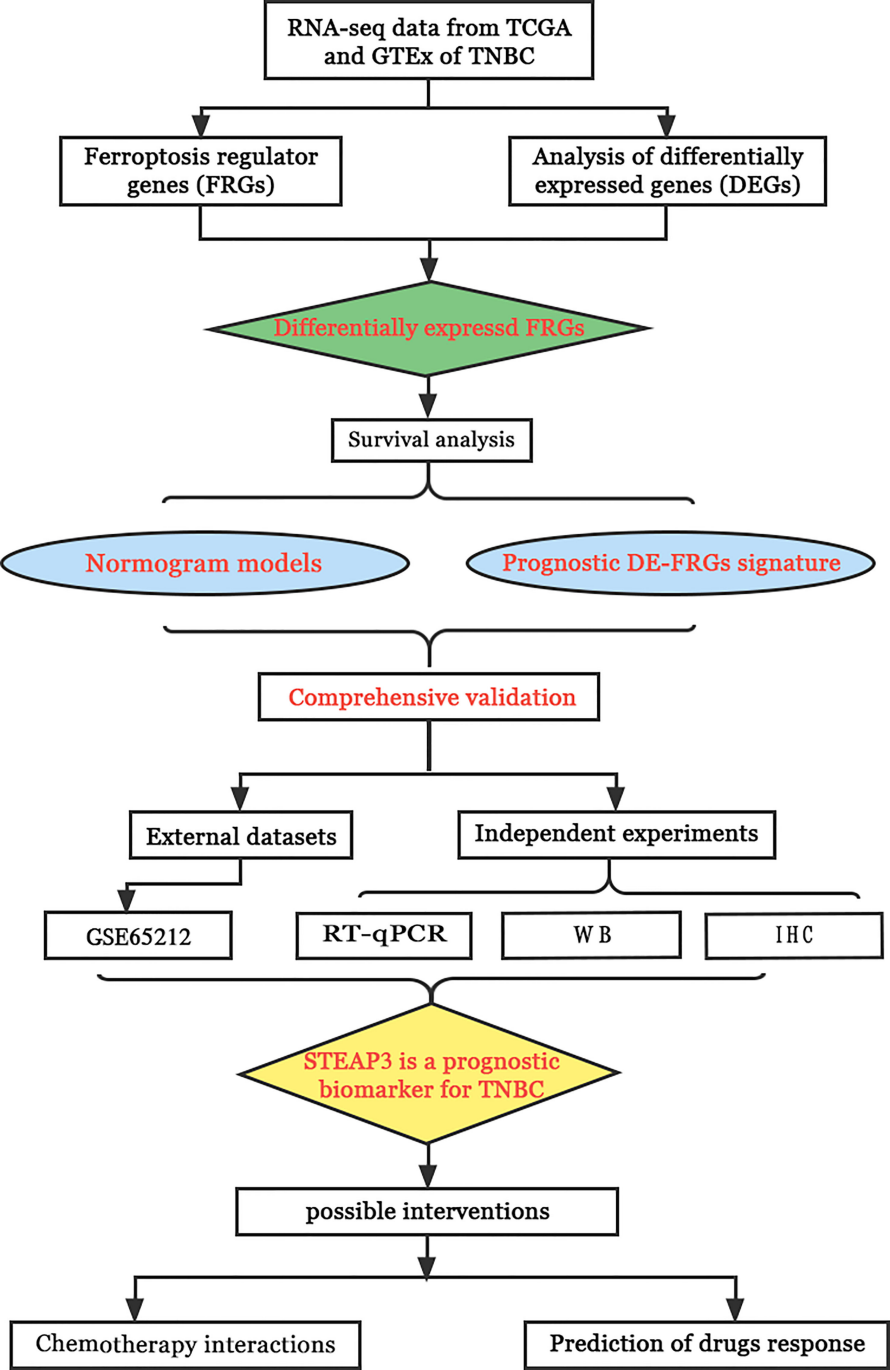
The schematic representation of the study workflow.

The current work was designed to fill this knowledge gap by analyzing the patterns of ferroptosis regulator gene (FRG) expression in TNBC utilizing information from TCGA database. The LASSO Cox regression model was then used with identified DE-FRGs to establish a TNBC-related FRG signature. Furthermore, the association between this signature and the TIME was defined, and the significance of this signature was confirmed in an independent patient cohort from the GEO. The expression of core FRGs in TNBC patient clinical samples and cell lines was subsequently analyzed to validate and expand upon these results. The workflow of the study is shown in **Figure 1**.

## Materials and methods

### Data sources

RNA-sequencing (RNA-seq) data and clinical information of 190 TNBC tissue samples (“ER- AND PE- AND HER2-”) and 572 normal tissue samples (113 in TCGA, 459 in GTEx) were retrieved from the TCGA (https://portal.gdc.com) and GTEx (https://commonfund.nih.gov/gtex) databases. Moreover, transcriptomic expression data from the external GSE65212 dataset (*n*_TNBC_=41, *n*_control_=11) were downloaded from the GEO database. **Table 1** compiles baseline data for TNBC patients included in the present investigation.

**Table 1:** The baseline clinical characteristics of the TNBC patients from TCGA used in this study.

Variables		N(%)
Survival tatus	Alive	165(86.84)
	Dead	25(13.16)
Age (Mean ± SD)		55.6±12.5
Gender	FEMALE	190(100.00)
Race	AMERICAN INDIAN	1(0.53)
	ASIAN	16(8.42)
	BLACK	57(30.00)
	WHITE	108(56.84)
	Unknown	8(4.17)
T	T1-T2	166(87.37)
	T3-T4	23(12.11)
	Unknown	1(0.53)
N	N0	115(60.53)
	N1	47(24.74)
	N2	16(8.42)
	N3	10(5.26)
	Unknown	2(1.05)
M	M0	166(87.37)
	M1	1(0.53)
	Unknown	23(12.10)
Stge	I	31(16.32)
	II	121(63.68)
	III	33(17.37)
	Unknown	5(2.63)
Chemotherapy	Chemotherapy	114(60.00)
	Other	76(40.00)

A total of 259 genes were defined as FRGs as per the FerrDb Version 2 (http://www.zhounan.org/ferrdb/). These FRGs were divided into drivers, suppressors, and markers. After excluding duplicate FRGs from these categories, 259 FRGs were used for the subsequent analyses.

False discovery rate (FDR)< 0.05 and |Log_2_ (Fold Change)| > 2 were used as a criterion for differential expression, and DE-FRGs were found using the R limma program. These analyses were carried out using R (v 4.0.3, 2020).

### Survival analyses

Using the R “survival” and “survminer” packages the overall survival (OS) of TNBC patients expressing low or high levels of particular DE-FRGs of interest was evaluated using a Kaplan-Meier procedure. Univariate and multivariate Cox regression analyses were used to examine the utility of DE-FRGs as predictors of patient OS, with data being compared with hazard ratios (HRs) and 95% confidence intervals (CIs) and log-rank tests, with *P<* 0.05 as the significance threshold.

### Nomogram development and validation

A prognostic Nomogram capable of predicting the 1-, 3-, and 5-year OS of patients in the TCGA-TNBC cohort was developed by initially identifying prognostic factors through Cox analyses. Age, pTstage, pNstage, pMstage were included into the Cox regression analyses. The other clinical variables were excluded due to their missing values. Concordance index (C-index) values ranging from 0.5 (poor) to 1.0 (perfect) were used to evaluate the performance of the resulting Nomogram. A calibration plot was further used to validate the accuracy of the Nomogram using the “rms” and “cmprsk” R packages, with *P*< 0.05 as the significance threshold.

### Establishment of a prognostic DE-FRG signature for TNBC

The effectiveness of DE-FRGs as potential prognostic biomarkers in TNBC patients was evaluated using a LASSO Cox analysis, and an ideal predictive risk signature model was created. The penalty parameter (λ) was determined using a minimum of 10-fold cross-validation. Analyses were conducted using the R survival package. Risk Score values were calculated based on the expression of all prognostic DE-FRGs included in this signature and the corresponding coefficient values using the following formula:


$$Risk\;score=\sum_{i=1}^{N}\left(Exp_{i}\times Coe_{i}\right)$$

Where ‘Expi’ corresponds to relative DE-FRG expression in this signature for patient ‘i’ and ‘Coefi’ is the Cox coefficient for DE-FRG*_i_*.

Optimal risk score cut-off values for the established DE-FRG signature were established with the “maxstat” package, with the minimum number of sample groups being > 25% and the maximum number being< 75%. Patients were divided into low- and high-risk groups based on these criteria. Log-rank tests and the “survfit” package were used to assess differences in prognostic outcomes between these groups.

The relationship between the established DE-FRG signature and levels of immune cell infiltration (B cells, CD4+ T cells, CD8+ T cells, neutrophils, macrophages, and dendritic cells) was examined with the Tumor Immune Estimation Resource database (TIMER, https://cistrome.shinyapps.io/timer/)

### Identification of prognostic FRGs in TNBC

The primary prognostic FRG predictive of TNBC patient OS was discovered using the DE-FRG signature and Nomogram models used in this study as *STEAP3*. Because of this, *STEAP3* was the main focus of later functional and validation analyses.

### Prediction of chemotherapeutic drug responses

A publicly available pharmacogenomics database (GDSC, https://www.cancerrxgene.org/) was used to predict the responses of individual TNBC samples to 7 different chemotherapeutic drugs (5-Fluorouracil, Cisplatin, GSK1904529A, AS601245, XMD8-85, Gefitinib, and Sorafenib). The R “pRRophetic” package was used for all studies, and the half-maximal inhibitory concentration (IC50) values were determined using a ridge regression method. Using the GDSC training set, 10-fold cross-validation was used to evaluate the predictive accuracy. Mean values were supplied for any duplicate gene names in the used datasets. In the datasets used, mean values were presented for any occurrences of duplication gene names. The “combat” and “allSolidTumours” packages were used to remove batch effects. All analyses were carried out in R 4.0.3 and a website tool.

### Cell culture and sample collection

The control MCF-10A human breast cell line, the MDA-MB-231, BT-549, and MDA-MB-468 TNBC cell lines, and the MCF-7, T-47D, BT-474 non-TNBC cell lines (Procell Life Science&Technology Co.,Ltd.Wuhan,China) were cultured in DMEM (Sparkjade, Shandong, China) containing 10% fetal bovine serum (FBS, PAN, Germany) and 1% penicillin-streptomycin in a humidified 37°C 5% CO_2_ incubator. Furthermore, 6 TNBC patient tissue samples were acquired from patients treated at The Department of Oncology of The Affiliated Yantai Yuhuangding Hospital of Qingdao University, China. All experiments were performed three times. Yantai Yuhuangding Hospital’s Institutional Review Board approved the trial, and all patients provided informed consent.

### RT-qPCR

Following the manufacturer’s instructions, RNA was extracted from cells using Trizol (Sparkjade, Shandong, China), and 0.5 μg of RNA per sample was then used to create cDNA using the SPARK script II RT Plus Kit (Sparkjade, Shandong, China). SYBR Green qPCR Mix kit with ROX (Sparkjade, Shandong, China) was subsequently used for qPCR analyses, and relative gene expression was assessed *via* the 2^-ΔΔCT^ method (27). GAPDH served as a normalization control. All samples were analyzed in triplicate, and experiments were independently repeated three times. Utilized primers were as follows: *STEAP3* (Forward: 5’- CTGGCAGTCAAGCAGGTCTTG -3’; Reverse: 5’- TTGAGCGAGTTTGCAATGGA -3’); *GAPDH* (Forward: 5’CATGTTCGTCATGGGTGTGAA-3’; Reverse: 5’-GGCATGGACTGTGGTCATGAG-3’).

### Western immunoblotting

After lysing tissue and cell samples using RIPA Lysis buffer supplemented with phosphatase inhibitors, the protein concentrations were determined using a BCA assay kit. Equal protein amounts were then separated *via* 12.5% SDS-PAGE, transferred onto PVDF blots, and incubated with rabbit anti-*STEAP3* (#55240,1:1000, Bioss, Beijing, China) or mouse anti-GAPDH (1:3000, Affinity, Shanghai, China) overnight at 4°C. Blots were then probed for 1 h with an HRP-linked secondary antibody (1:10,000, Affinity, Shanghai, China) at room temperature, after which enhanced chemiluminescence detection of protein bands was performed. ImageJ was used for densitometric analyses. GAPDH served as a loading control.

### Immunohistochemical staining

From patients who had given informed consent, a total of 35 breast tissue samples, including 23 control and TNBC, 12 control and non-TNBC (*n*_Luminal A_ =3, *n*_Luminal B_ =5, *n*_Her2-enriched_ =4) were taken for IHC staining. The 4 mm tissue sections were mounted on glass slides, deparaffinized with xylene, rehydrated with an ethanol gradient, and then heated to a high temperature for antigen retrieval. After cooling and washing, samples were treated with 3% H_2_O_2_ to quench endogenous peroxidase activity. Following three rinses with PBS, samples were blocked for 10 min in calf serum, followed by overnight incubation with polyclonal rabbit anti-STEAP3 (#55240, 1:200, Bioss, Beijing, China) at 4°C. A suitable primary antibody was used to probe samples for 30 to 40 minutes at room temperature. Sections were then dried and photographed under a light microscope. Two pathologists blinded to sample sources analyzed all IHC staining results, and staining intensity was assessed semi-quantitatively.

### Statistical analyses

Data are reported as means ± standard errors of the means $\left(\bar{x}\pm SEM\right)$ , and were compared using SPSS 25.0 *via* two-tailed Student’s t-tests or non-parametric tests when normally and non-normally distributed, respectively. The significance threshold for these analyses was α = 0.05.

## Results

### TNBC-related FRG identification

**Figure 2:**
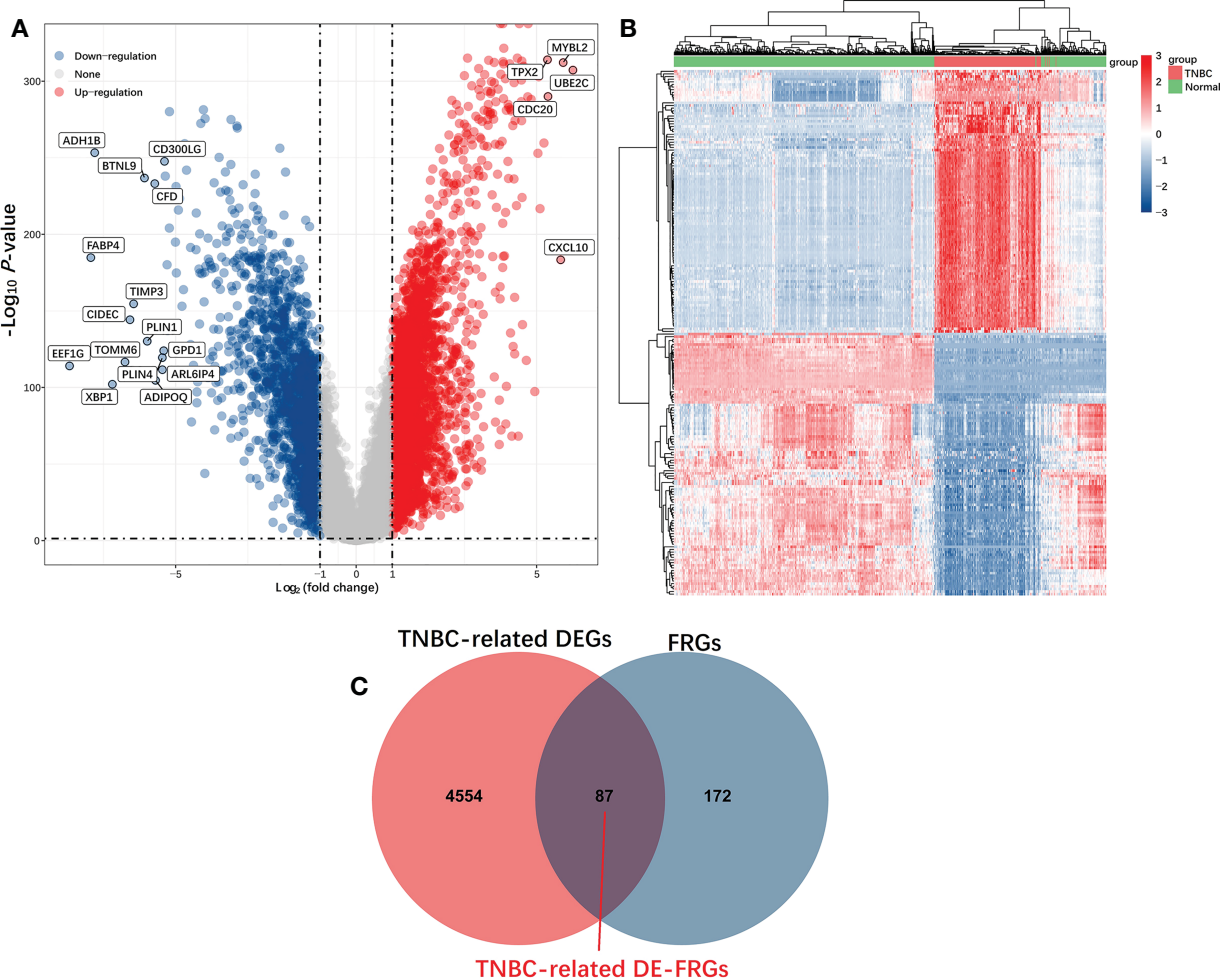
Identification of TNBC-related DE-FRGs in the TCGA database. DE-FRGs that were differentially expressed FRGs between TNBC tumors and healthy tissues (FDR < 0.05, |Log2 (Fold Change)| > 2) were presented in the form of Volcano plots **(A)**, in which blue and red dots respectively correspond to up-regulated and downregulated DE-FRGs; Heat maps **(B)**, in which each dot and its color (Red is high-expression, blue is low-expression) indicate the expression value of each DE-FRGs in different samples, the greater the expression level, the darker the color. **(C)** Venn diagrams were used to identify TNBC-associated FRGs.

The current analysis comprised 259 previously constituted FRGs in total. When comparing TNBC tumors and healthy tissue samples (87/259, 33.59%), 87 of them were discovered to be differentially expressed (FDR< 0.05, FC > 2) (**Figure 2**).

### Identification and assessment of prognostic TNBC-associated FRGs

**Figure 3:**
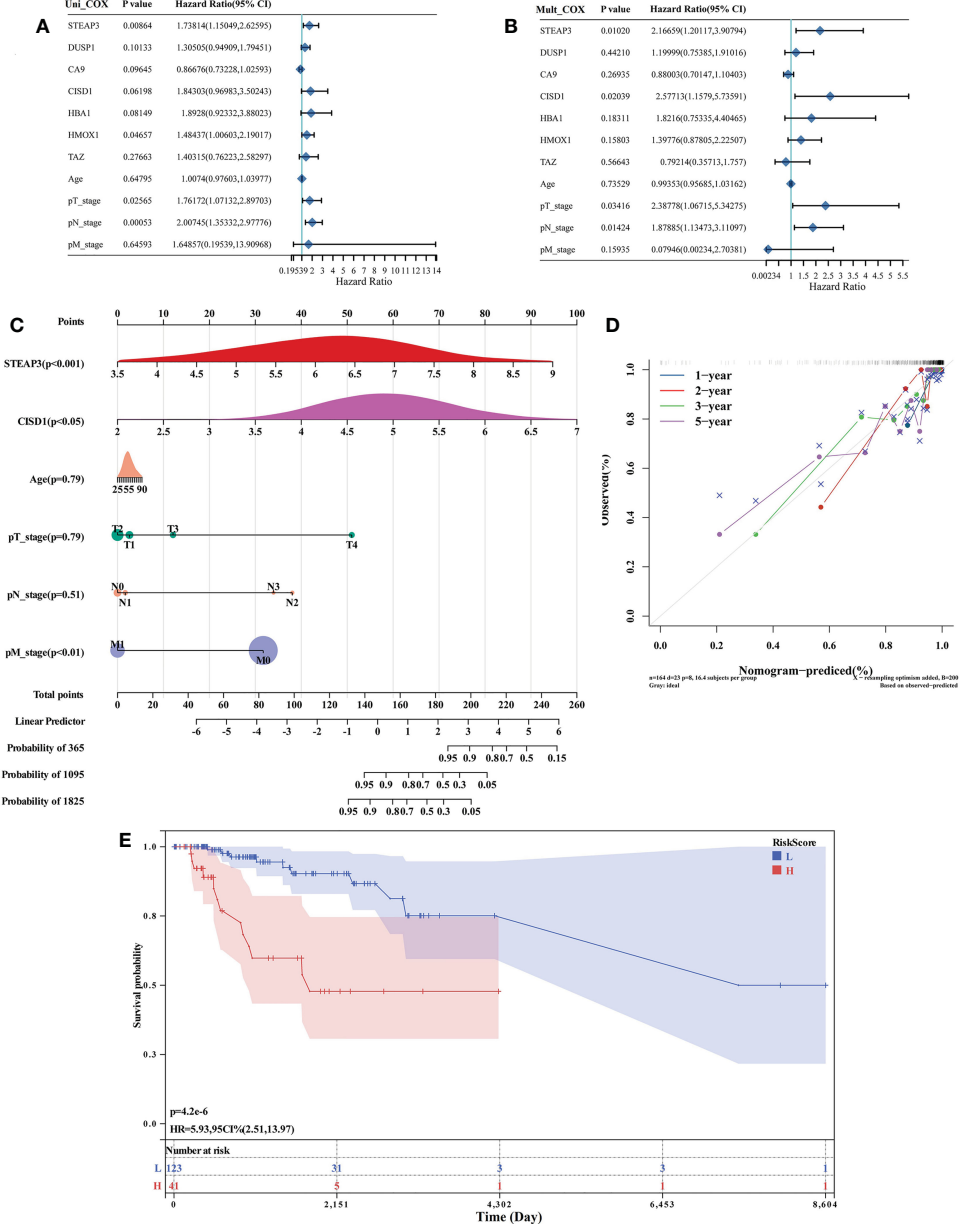
Identification and assessment of prognostic TNBC-associated FRGs. **(A, B)** Forest plot-based identification of TNBC patient risk factors identified through univariate **(A)** and multivariate **(B)** Cox regression analyses. **(C)** A nomogram was established based on multivariate Cox regression analysis results. **(D)** Calibration plot for the established Nomogram. **(E)** Evaluation of the developed Nomogram based on Kaplan-Meier OS curves.

Of the 87 identified DE-FRGs related to TNBC, 7 were significantly correlated with patient OS in Kaplan-Meier analyses (*CA9, CISD1, STEAP3, HMOX1, DUSP1, TAZ, HBA1*). Forest plots indicated that *CISD1* and *STEAP3* were associated with the highest level of risk for TNBC patients, as they exhibited HRs > 1 in univariate and multivariate Cox regression analyses (P< 0.05) (**Figures 3A, B**). These results were then used to establish a nomogram incorporating four factors associated with TNBC patient prognosis (*CISD1*, *STEAP3*, pTstage, pNstage). In this model, points were assigned for each risk factor and then summed to produce an overall value, with higher total points corresponding to worse patient OS. The C-index for this model was 0.87 (95% CI: 0.82-0.93; *P* = 5.46×10^-42^), and a calibration plot confirmed that this Nomogram exhibited satisfactory utility for use in clinical practice (**Figures 3C, D**). The R ‘maxstat’ package was next employed to calculate optimal Risk Score cut-off values as detailed in the Materials and Methods section, ultimately defining an ideal Risk Score threshold of 0.9395. The prognosis of the patients in these groups was compared using log-rank tests using OS values, which were used to stratify TNBC patients into two groups based on whether they were above or below this cut-off value. These analyses indicated that patients in the high-Risk Score cohort exhibited a shorter OS than patients in the low-Risk Score cohort (*P* = 4.2×10^-6^, **Figure 3E**).

### Establishment of a TNBC ferroptosis-related prognostic gene signature

**Figure 4:**
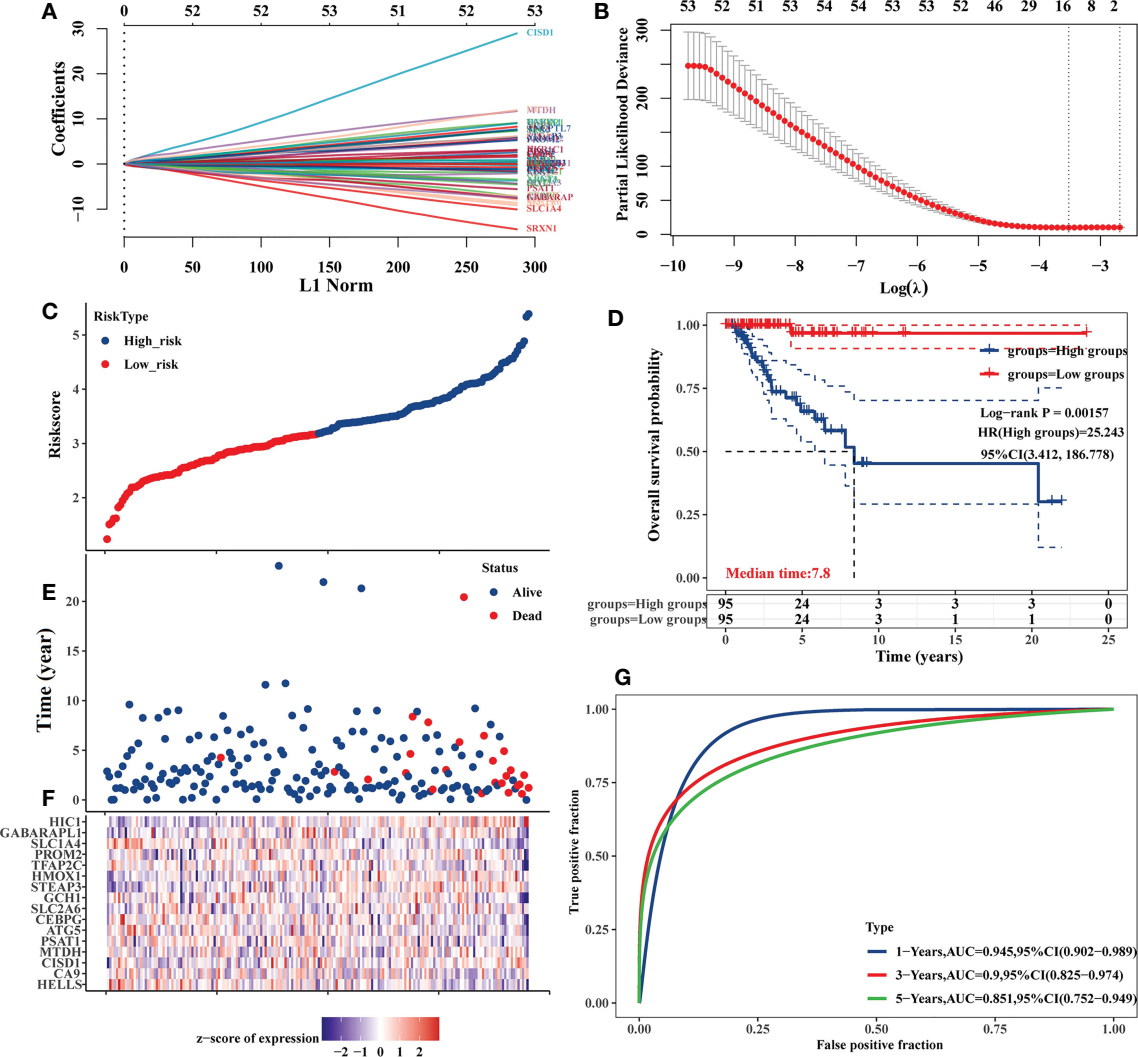
Development of an 87 DE-FRG-based prognostic risk signature in the TCGA-TNBC cohort. **(A)** LASSO coefficient profiles for 87 DE-FRGs. **(B)** LASSO regression analyses with 10-fold cross-validation yielded 16 prognostic DE-FRGs based on a minimum λ value. **(C, E)** OS distributions, OS status, and risk scores for patients in the TCGA-TNBC cohort. **(D)** Kaplan-Meier curves corresponding to the OS of TCGA-TNBC patients stratified into low- and high-risk groups. **(F)** Z-scores corresponding to the expression of the 16 prognostic DE-FRGs included in the established risk signature. **(G)** AUC values for time-dependent ROC curves were employed to assess the predictive utility of prognostic signature-derived risk scores.

The expression of the 87 DE-FRGs discovered above was then used to create a prediction model using a LASSO Cox regression technique. Based on the expression of particular genes (identified by gene name), the Risk Score for this model was calculated as follows: Risk score=(-0.0863) × *HELLS* + (-0.0361) × *CA9* + (0.4763) × *CISD1* + (0.3359) × *MTDH* + (-0.0291) × *PSAT1* + (-0.1691) × *ATG5* + (-0.0738) × *CEBPG* + (-0.0423) × *SLC2A6* + (-0.0698) × *GCH1* + (0.3107) × *STEAP3* + (0.0688) × *HMOX1* + (-0.1375) × *TFAP2C* + (0.0344) × *PROM2* + (-0.318) × *SLC1A4* + (-0.0162) × *GABARAPL1* + (0.4724) × *HIC1* (λ_min_=0.0296) (**Figures 4A, B**). Patients from the TCGA-TNBC cohort were stratified into low-risk and high-risk cohorts (n=95 each) based on median Risk Score values computed with this model (**Figures 4C–F**). Kaplan-Meier analyses indicated that the OS of high-risk patients was significantly worse than that of low-risk patients (*P* = 0.00157) (**Figure 4D**). AUC values of 0.945, 0.900, and 0.851 at 1, 3, and 5 years, respectively, from time-dependent ROC curve assessments of the predictive utility of this model, further confirm its good utility as a predictor of TNBC patient OS (**Figure 4G**).

**Figure 5:**
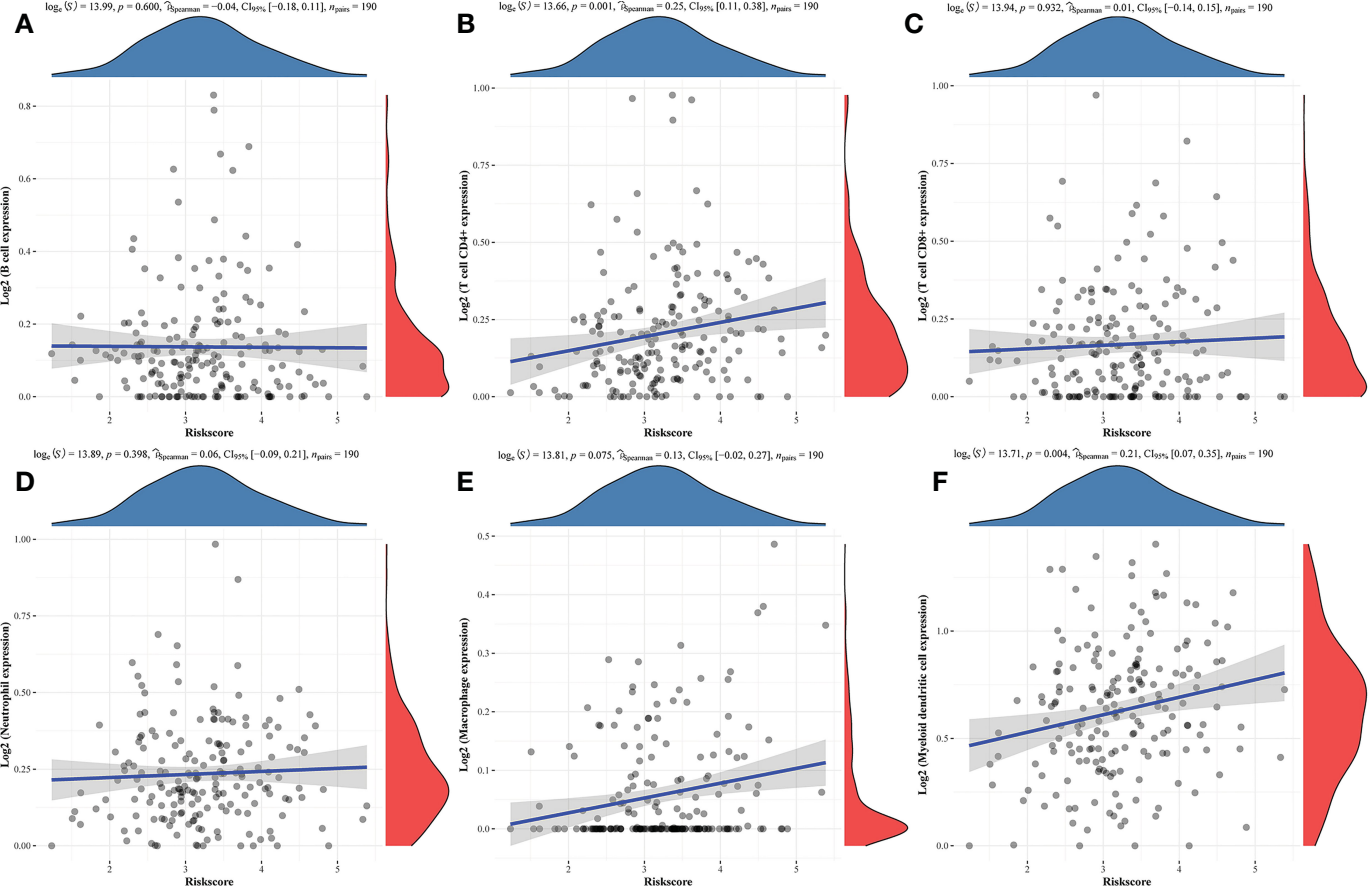
Correlations between prognostic risk score values and immune cell infiltration. **(A-F)** Correlations between predictive risk scores and the six indicated immune cell types.

Additionally, the TIMER database used correlation studies of risk scores and immune cell infiltration levels to investigate the clinical significance of this signature in TNBC patients (**Figure 5**). The results of this analysis indicated that these prognostic Risk Score values were positively correlated with the infiltration of CD4+ T cells (P = 0.001, **Figure 5B**) and myeloid dendritic cells (P = 0.004, **Figure 5F**).

### Overall survival analyses

**Figure 6:**
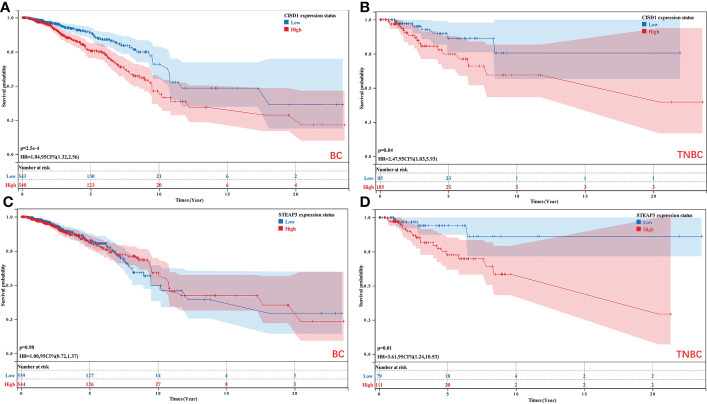
Analyses of the relationship between *CISD1* and *STEAP3* expression and the survival of BRCA and TNBC patients. **(A, B)** The relationship between the expression of *CISD1* and the OS of BC (*P*<0.05) and TNBC patients (*P*<0.05). **(C, D)** The relationship between the expression of *STEAP3* and the OS of BC (*P*>0.05) and TNBC patients (*P*<0.05).

The nomogram and risk signature models established the above-identified *CISD1* and *STEAP3* predictors of TNBC patient outcomes. Accordingly, Kaplan-Meier analyses were performed for these two genes in breast cancer (BRCA) and TNBC patients. While *CISD1* expression was significantly associated with the OS of both BRCA and TNBC patients (*P*<0.05), *STEAP3* expression was specifically associated with TNBC patient OS (*P*<0.05) (**Figure 6**).

### Validation of *STEAP3* expression in an independent TNBC patient cohort

**Figure 7:**
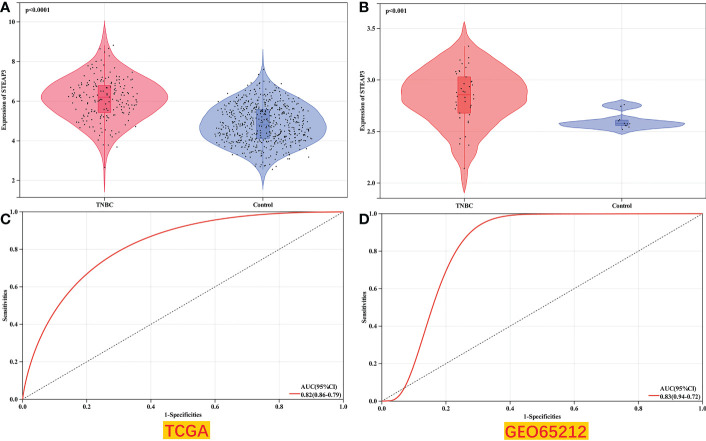
Validation of TNBC patient *STEAP3* expression levels in GEO datasets. **(A, B)** *STEAP3* expression levels were compared between TNBC and normal tissue samples in the selected TCGA (*P*<0.0001) and GEO (*P*<0.001) datasets. **(C, D)** AUC analyses for the TCGA and GEO datasets.

For external validation, the GSE65212 dataset was retrieved from the GEO database to verify the differential expression of *STEAP3* in TNBC in a different patient cohort. Compared to healthy control samples, this dataset’s analyses revealed that *STEAP3* was significantly up-regulated in TNBC patients (P< 0.05, **Figure 7**).

### Analyses of *STEAP3* expression in clinical samples and cell lines

**Figure 8:**
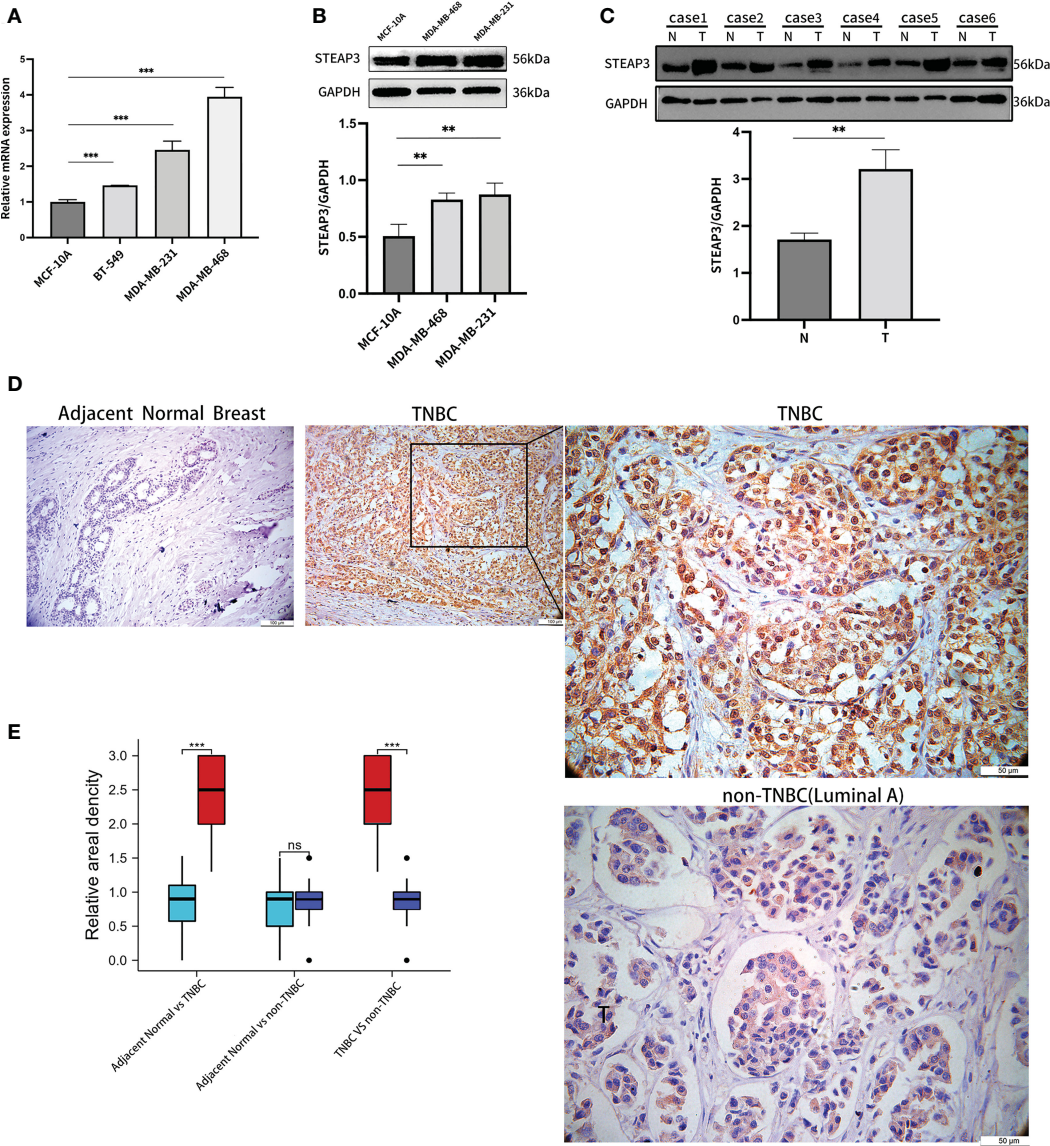
Validation of *STEAP3* expression in breast cancer cell lines and tissue samples. **(A)** *STEAP3* mRNA levels were assessed in the control MCF-10A cell line and the MDA-MB-231, BT-549, and BT-468 TNBC cell lines. **(B, C)** STEAP3 levels were detected *via* Western immunoblotting in the MCF-10A, MDA-MB-468, and MDA-MB-231 cell lines and in six pairs of TNBC (T) and adjacent normal (N) tissue samples from patients. **(D)** Representative IHC staining results for STEAP3 in adjacent normal tissues (Scale bar: 100 μm) and TNBC samples (Scale bars:100μm and 50 μm), and non-TNBC(Luminal A) samples (Scale bar: 50μm). **(E)** Quantitative data from IHC staining results for *STEAP3* expression in 23 TNBC vs adjacent normal breast,12 non-TNBC vs adjacent normal breast and 23 TNBC vs 12 non-TNBC are shown. (**P<0.01, ***P< 0.001, ^ns^ P>0.05).

Subsequently, *STEAP3* mRNA levels were determined using RT-qPCR in the control MCF-10A human breast cell line and in the MDA-MB-231, MDA-MB-468, and BT-549 TNBC cell lines to validate the findings mentioned above (**Figure 8A**). These analyses revealed significantly increased *STEAP3* expression in all three TNBC cell lines relative to MCF-10A cell lines, consistent with the above bioinformatics analyses. In contrast, no significant *STEAP3* expression was observed in the non-TNBC MCF-7, T-47D, and BT-474 cell lines relative to MCF-10A cells (**Figure S1**). *STEAP3* protein levels were also significantly increased in MDA-MB-468 and MDA-MB-231 cells relative to MCF-10A cells, with comparable findings in 6 pairs of matched TNBC patient tissue samples (**Figures 8B, C**). And no significant *STEAP3* expression was observed in the non-TNBC MCF-7 and BT-474 cell lines relative to MCF-10A cells (**Figure S2**). IHC staining was additionally used to assess *STEAP3* protein levels in Normal vs TNBC, Normal vs non-TNBC and TNBC vs non-TNBC, revealing significantly increased *STEAP3* expression in 23 TNBC tumor tissue samples relative to matched paracancerous samples but no increase *STEAP3* expression was observed in the non-TNBC and its control group. And *STEAP3* expression in TNBC was significantly higher than non-TNBC (**Figures 8D, E**).These findings show that, compared to relevant control samples, TNBC tumor cells and tissue exhibit a considerable up-regulation of *STEAP3* at the mRNA and protein levels.

### Correlations between TNBC patient OS, *STEAP3* expression, and other clinical parameters

A multivariate logistic regression approach was next used to examine the relationship between *STEAP3* and TNBC patient OS using a multivariate logistic regression approach. These analyses revealed increased *STEAP3* levels as an independent risk factor associated with the OS of patients with TNBC (OR=5.410, 95%CI: 2.040-14.348). Chemotherapy and *STEAP3* interacted with TNBC patient prognosis (OR=0.482, 95%CI: 0.339-0.686). These findings demonstrated that high levels of *STEAP3* expression had a detrimental effect on TNBC patient OS, but chemotherapeutic therapy was sufficient to reverse this effect (**Table 2**).

**Table 2:** Interactions between *STEAP3* with chemotherapy on OS of TNBC.

Variables	*β*	*S.E.*	*Wald*	*P*	*OR*	*95% C.I. for OR*
*STEAP3*	1.688	0.498	11.509	0.001	5.41	2.040-14.348
*STEAP3* by chemotherapy	-0.729	0.180	16.418	<0.001	0.482	0.339-0.686

### *STEAP3* expression levels predict TNBC patient responses to chemotherapeutic treatment

**Figure 9:**
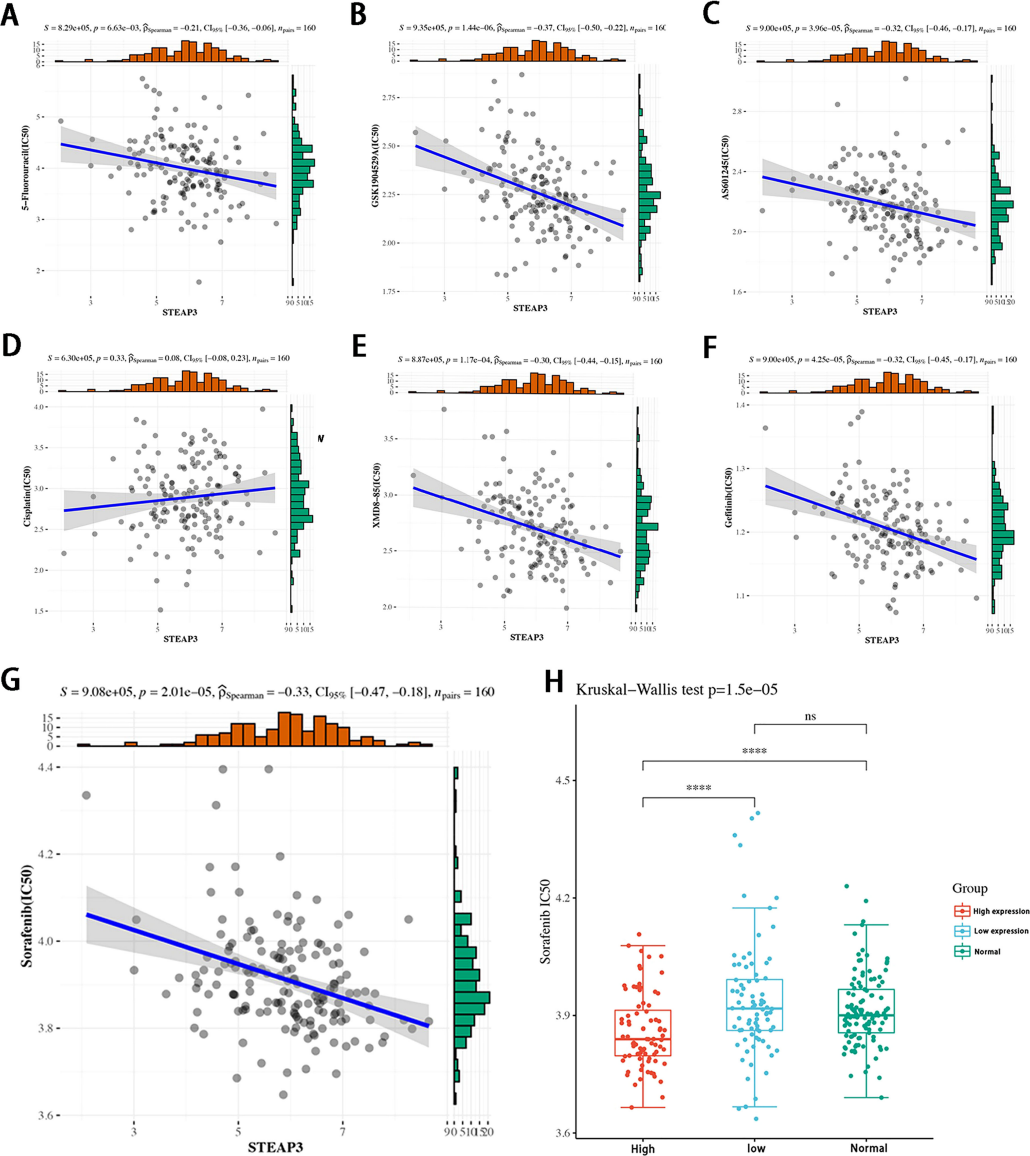
Correlations between STEAP3 expression and drug sensitivity in patients with TNBC. **(A-F)** The expression of STEAP3 and IC50 values correspond to 5−Fluorouracil, GSK1904529A, AS601245, XMD8−85, and Gefitinib in TNBC patients included in the TCGA-TNBC cohort (P < 0.05). **(G, H)** Correlations between *STEAP3* expression and Sorafenib IC50 values with further details regarding expression in the low, high, and normal groups (P<0.01). (****P< 0.0001, ns P>0.05).

While *STEAP3* expression levels were not related to cisplatin sensitivity (P=0.33), they were significantly negatively correlated with the sensitivity of patients in the TCGA-TNBC cohort to 5-Fluorouracil, GSK1904529A (IGF1R inhibitor), AS601245 (JNK inhibitor), XMD8−85 (Erk5 inhibitor), Gefitinib, and Sorafenib (P < 0.01, **Figures 9A–G**). The expression levels of *STEAP3* and the sensitivity of Sorafenib in patient groups with low and high expression and healthy controls were also evaluated. The IC50 values between the high and low expression groups differed significantly. However, there was no discernible difference in these values between the low expression and healthy control sample groups (**Figure 9H**). These findings imply that *STEAP3* expression in TNBC patients may be useful as a predictor of patient responses to a variety of small molecule medications and pathway inhibitors.

## Discussion

TNBC cases are account for 15-20% of all breast cancer patients yet are associated with higher recurrence and metastasis rates than other subtypes positive for these receptors, with the poorest corresponding patient prognosis in clinical settings (28, 29). However, the genetic variables that cause TNBC recurrence remain unknown. Efforts to explain the molecular aetiology of TNBC formation, progression, chemoresistance, and recurrence can potentially aid drug development efforts.

Ferroptosis is iron-dependent cell death independent of apoptosis, necroptosis, and autophagy-related cell death (16, 30). Mechanistically, ferroptosis death occurs due to severe ROS-induced lipid peroxidation within cells and iron overload (30), which is increasingly well-studied in many human diseases in recent years, including in TNBC (31, 32). However, this ferroptosis process remains extremely complex and is regulated by a diverse of biomolecular intermediaries and metabolites such that the precise mechanisms driving ferroptosis remain incompletely understood.

*STEAP3* encodes a multi-pass membrane metalloreductase that serves as an iron transporter capable of reducing Fe^3+^ and Cu^2+^ cations. Mechanistically, STEAP3 may regulate downstream p53 responses and apoptotic cell death. Deficient *STEAP3* expression can contribute to anaemia. Several alternative splice variants of *STEAP3* exist. The findings of this study reveal that *STEAP3* overexpression is associated with worse OS outcomes in TNBC patients, possibly due to reduced Fe3^+^ transport in these patients. *STEAP3* has previously been shown to play a role in a range of malignant solid tumor types (33–36), yet its role in TNBC has yet to be established. While *STEAP3* was unrelated to any improvement in overall breast cancer patient OS, other studies have found a relationship between downregulation of the related *STEAP1*, *STEAP2*, and *STEAP4* proteins and improved outcomes (37). It was first discovered that TNBC had significantly higher levels of *STEAP3* expression, which was associated with a bad prognosis for the patient. Importantly, independent of the examined TCGA and GEO datasets, these changes in expression were verified at the mRNA and protein levels using TNBC cell lines and clinical samples. However, *STEAP3* expression in non-TNBC cell lines, non-TNBC tissue and overall breast cancer patient cohorts indicated that this gene is not up-regulated or linked to the OS of these patients (**Figures S1–S3**, **8D, E**). As a result, *STEAP3* overexpression may represent a TNBC cell-specific biomarker. These studies confirmed the proposed predictive risk signature model for TNBC while indicating that *STEAP3* overexpression is related to a lower TNBC patient survival rate.

Further analyses were performed to conduct a cursory exploration of the mechanisms whereby *STEAP3* may influence TNBC patient OS based on TIME composition, chemotherapy interactions, and drug sensitivity profiles. Breast cancer is generally considered a “cold tumor” with low immunogenicity compared to melanoma, renal cancer and lung cancer. Although the results of the IMpassion130 and KEYNOTE-522 studies show that immunotherapy can significantly affect TNBC patients, the overall improvement in their prognosis remains suboptimal following treatment (38, 39). Studies have shown that dentritic cell and CD4+T cell are involved in tumor progression (40, 41). In this analysis, prognostic Risk Scores were also positively correlated with CD4+ T cells and myeloid dendritic cell infiltration, suggesting that these prognostic risks are may related to tumor immune regulation.

Understanding the mechanisms that cause ferroptosis better may open up new treatment options for TNBC and other disorders without effective therapy options. A possible involvement for ferroptosis cell death in the beginning and progression of this cancer type is suggested by the differential expression of 87 FRGs in TNBC. These included 29 (33.33%) TNBC suppressors, 25 (28.74%) TNBC drivers, and 42 (48.28%) TNBC markers, suggesting that ferroptosis plays diverse roles in the regulation of TNBC and underscoring the importance of further work clarifying the particular mechanisms underlying the interplay between this form of cell death and this deadly disease. At present, reliable inducers of ferroptosis remain an active area of research and drug development interest owing to the complexity of this process. These drugs may target the transporters and enzymes necessary for iron, amino acid, and lipid metabolism, as well as redox balance (1, 32). As a result, ferroptosis holds considerable potential in treating cancer cells resistant to apoptosis in future.

*STEAP3* expression and chemotherapeutic treatment affect TNBC patient outcomes, suggesting that chemotherapy can reverse the adverse impacts of high levels of *STEAP3* expression on TNBC patient OS. Therefore, these TNBC patients may constitute significant high-priority candidates for chemotherapy treatment. As an alternative to standard cytotoxic chemotherapeutic treatments, there is an urgent need to develop effective drugs to treat TNBC. In this study, it was predicted that TNBC patients expressing higher levels of *STEAP3* would be more sensitive to a variety of small molecule pathway inhibitor drugs targeting the IGF1R, JNK, ERK5, EGF, and EGFR pathways, with Sorafenib sensitivity being particularly pronounced as a function of *STEAP3* expression. Prior *in vitro* work has shown that Gefitinib-based EGFR blockade may be therapeutically beneficial in TNBC patients. Combining Gefitinib with ERK pathway inhibitors is linked to reduced TNBC cell proliferation (42, 43). Here, we found numerous small-molecule pathway inhibitor drugs were anticipated to be more successful in patients with higher levels of *STEAP3* expression, these findings may be useful for researchers and physicians contemplating the usage or selection of cytotoxic therapies to treat TNBC.

There are two key limitations to the present study. These analyses were primarily based on retrospectively analyzed data from public databases. As such, additional prospective multicenter verification will be essential. Accordingly, our centre’s cell-based validation studies of sorafenib sensitivity, ferroptosis sensitivity and the functional experiments of *STEAP3* are currently being performed. Secondly, this study only focused on FRGs, and the relationships between these genes and other potentially relevant biomarkers were not assessed.

In conclusion, the unique FRG model developed here can be used to forecast the prognosis of TNBC patients. Future efforts to more reliably and successfully treat this lethal breast cancer subtype will be built on the realization that the altered expression of STEAP3 in these individuals may have ramifications for overall survival and therapeutic strategies.

## Data availability statement

The datasets presented in this study can be found in online repositories. The names of the repository/repositories and accession number(s) can be found in the article/**Supplementary Material**.

## Ethics statement

The studies involving human participants were reviewed and approved by Yuhuangding Hospital’s Institutional Research Ethics Committee. The patients/participants provided their written informed consent to participate in this study.

## Author contributions

LY, JL, LB, HQ and PS conceived, LY designed, analyzed the data and wrote the manuscript. JL and HQ revised the manuscript. LB participated in reviewing pathology. All authors read and approved the final manuscript. All authors contributed to the article and approved the submitted version.

## References

[1] Siegel RL Miller KD Fuchs HE Jemal A. Cancer statistics 2022. CA Cancer J Clin (2022) 72:7–33. doi: 10.3322/caac.21708 35020204

[2] Halbony H Salman K Alqassieh A Albrezat M Hamdan A Abualhaija’a A. Breast cancer epidemiology among surgically treated patients in Jordan: A retrospective study. Med J Islam. Repub. Iran (2020) 34:73. doi: 10.34171/mjiri.34.73 33306068 PMC7711030

[3] Daily K Douglas E Romitti PA Thomas A. Epidemiology of *De novo* metastatic breast cancer. Clin Breast Cancer (2021) 21:302–8. doi: 10.1016/j.clbc.2021.01.017 33750642

[4] Ezeome ER Yawe KT Ayandipo O Badejo O Adebamowo SN Achusi B. The African female breast cancer epidemiology study protocol. Front Oncol (2022) 12:856182. doi: 10.3389/fonc.2022.856182 35494056 PMC9044037

[5] Zhou Y Yang J Chen C Li Z Chen Y Zhang X. Polyphyllin-induced ferroptosis in MDA-MB-231 triple-negative breast cancer cells can be protected against by KLF4-mediated upregulation of xCT. Front Pharmacol (2021) 12:670224. doi: 10.3389/fphar.2021.670224 34040532 PMC8141818

[6] Wei Y Zhu Z Hu H Guan J Yang B Zhao H. Eupaformosanin induces apoptosis and ferroptosis through ubiquitination of mutant p53 in triple-negative breast cancer. Eur J Pharmacol (2022) 924:174970. doi: 10.1016/j.ejphar.2022.174970 35469839

[7] Yao X Xie R Cao Y Tang J Men Y Peng H. Simvastatin induced ferroptosis for triple-negative breast cancer therapy. J Nanobiotechnolog (2021) 19 (1):311. doi: 10.1186/s12951-021-01058-1 PMC8502296 34627266

[8] Ding Y Chen X Liu C Ge W Wang Q Hao X. Identification of a small molecule as inducer of ferroptosis and apoptosis through ubiquitination of GPX4 in triple negative breast cancer cells. J Hematol Oncol (2021) 14:19. doi: 10.1186/s13045-020-01016-8 33472669 PMC7816340

[9] Du J Wang L Huang X Zhang N Long Z Yang Y. Shuganning injection, a traditional Chinese patent medicine, induces ferroptosis and suppresses tumor growth in triple-negative breast cancer cells. Phytomedicine (2021) 85:153551. doi: 10.1016/j.phymed.2021.153551 33827043

[10] Haffty BG Yang Q Reiss M Kearney T Higgins SA Weidhaas J. Locoregional relapse and distant metastasis in conservatively managed triple negative early-stage breast cancer. J Clin Oncol (2006) 24:5652–7. doi: 10.1200/JCO.2006.06.5664 17116942

[11] Kassam F Enright K Dent R Dranitsaris G Myers J Flynn C. Survival outcomes for patients with metastatic triple-negative breast cancer: Implications for clinical practice and trial design. Clin Breast Cancer (2009) 9:29–33. doi: 10.3816/CBC.2009.n.005 19299237

[12] Doval DC Sharma A Sinha R Kumar K Dewan AK Chaturvedi H. Immunohistochemical profile of breast cancer patients at a tertiary care hospital in new Delhi, India. Asian Pac. J Cancer Prev (2015) 16:4959–64. doi: 10.7314/APJCP.2015.16.12.4959 26163622

[13] Galluzzi L Humeau J Buque A Zitvogel L Kroemer G. Immunostimulation with chemotherapy in the era of immune checkpoint inhibitors. Nat Rev Clin Oncol (2020) 17:725–41. doi: 10.1038/s41571-020-0413-z 32760014

[14] Drago JZ Modi S Chandarlapaty S. Unlocking the potential of antibody-drug conjugates for cancer therapy. Nat Rev Clin Oncol (2021) 18:327–44. doi: 10.1038/s41571-021-00470-8 PMC8287784 33558752

[15] Singh DD Parveen A Yadav DK. Role of PARP in TNBC: Mechanism of inhibition, clinical applications, and resistance. Biomedicines (2021) 9:1512. doi: 10.3390/biomedicines9111512 34829741 PMC8614648

[16] Wu ZH Tang Y Yu H Li HD. The role of ferroptosis in breast cancer patients: A comprehensive analysis. Cell Death Discovery (2021) 7:93. doi: 10.1038/s41420-021-00473-5 33947836 PMC8097021

[17] Cao X Li Y Wang Y Yu T Zhu C Zhang X. Curcumin suppresses tumorigenesis by ferroptosis in breast cancer. PloS One (2022) 17:e0261370. doi: 10.1371/journal.pone.0261370 35041678 PMC8765616

[18] Zhang J Gao RF Li J Yu K Bi K. Alloimperatorin activates apoptosis, ferroptosis and oxeiptosis to inhibit the growth and invasion of breast cancer cells in vitro. Biochem Cell Biol (2022) 100(3):213–22. doi: 10.1139/bcb-2021-0399 35263194

[19] Tang W Xu F Zhao M Zhang S. Ferroptosis regulators, especially SQLE, play an important role in prognosis, progression and immune environment of breast cancer. BMC Cancer (2021) 21:1160. doi: 10.1186/s12885-021-08892-4 34715817 PMC8555209

[20] Li H Li L Xue C Huang R Hu A An X. A novel ferroptosis-related gene signature predicts overall survival of breast cancer patients. Biology (Basel) (2021) 10 (2):151. doi: 10.3390/biology10020151 33672990 PMC7917807

[21] Jin LY Gu YL Zhu Q Li XH Jiang GQ. The role of ferroptosis-related genes for overall survival prediction in breast cancer. J Clin Lab Anal (2021) 35:e24094. doi: 10.1002/jcla.24094 34741349 PMC8649350

[22] Lee N Carlisle AE Peppers A Pack SJ Doshi MB Spears ME. xCT-driven expression of GPX4 determines sensitivity of breast cancer cells to ferroptosis inducers. Antioxidants (Basel) (2021) 10:317. doi: 10.3390/antiox10020317 33672555 PMC7923775

[23] Zeitler L Fiore A Meyer C Russier M Zanella G Suppmann S. Anti-ferroptotic mechanism of IL4i1-mediated amino acid metabolism. Elife (2021) 10:e64806. doi: 10.7554/eLife.64806 33646117 PMC7946422

[24] Stockwell BR. Ferroptosis turns 10: Emerging mechanisms, physiological functions, and therapeutic applications. Cell (2022) 185:2401–21. doi: 10.1038/nature10933 PMC9273022 35803244

[25] Brown CW Amante JJ Chhoy P Elaimy AL Liu H Zhu LJ. Prominin2 drives ferroptosis resistance by stimulating iron export. Dev Cell (2019) 51:575–586.e4. doi: 10.1016/j.devcel.2019.10.007 31735663 PMC8316835

[26] Yu M Gai C Li Z Ding D Zheng J Zhang W. Targeted exosome- encapsulated erastin induced ferroptosis in triple negative breast cancer cells. Cancer Sci (2019) 110:3173–82. doi: 10.1111/cas.14181 PMC6778638 31464035

[27] Livak KJ Schmittgen TD. Analysis of relative gene expression data using real-time quantitative. PCR and the 2– ΔΔCT method. Methods (2001) 25(4):402–8. doi: 10.1006/meth.2001.1262 11846609

[28] Shah SP Roth A Goya R Oloumi A Ha G Zhao Y. The clonal and mutational evolution spectrum of primary triple-negative breast cancers. Nature (2012) 486:395–9. doi: 10.1038/nature10933 PMC3863681 22495314

[29] The Cancer Genome Atlas Network. Comprehensive molecular portraits of human breast tumours. Nature (2012) 490:61–70. doi: 10.1038/nature11412 23000897 PMC3465532

[30] Li Z Chen L Chen C Zhou Y Hu D Yang J. Targeting ferroptosis in breast cancer. biomark Res (2020) 8:58. doi: 10.1186/s40364-020-00230-3 33292585 PMC7643412

[31] Wang LL Luo J He ZH Liu YQ Li HG Xie D. STEAP3 promotes cancer cell proliferation by facilitating. nuclear trafficking of EGFR to enhance RAC1-ERK-STAT3 signaling in hepatocellular carcinoma. Cell Death Dis (2021) 12:1–12. doi: 10.1038/s41419-021-04329-9 34741044 PMC8571373

[32] Lin HY Ho HW Chang YH Wei CJ Chu PY. The evolving role of ferroptosis in breast cancer: Translational implications present and future. Cancers (Basel) (2021) 13:4576. doi: 10.3390/cancers13184576 34572802 PMC8466180

[33] Han M Xu R Wang S Yang N Ni S Zhang Q. Six-transmembrane epithelial antigen of prostate 3. predicts poor prognosis and promotes glioblastoma growth and invasion. Neoplasia (2018) 20:543–54. doi: 10.1016/j.neo.2018.04.002 PMC5994776 29730475

[34] Machlenkin A Paz A Bar Haim E Goldberger O Finkel E Tirosh B. Human CTL epitopes prostatic acid. phosphatase-3 and six-transmembrane epithelial antigen of prostate-3 as candidates for prostate cancer immunotherapy. Cancer Res (2005) 65:6435–42. doi: 10.1158/0008-5472.CAN-05-0133 16024648

[35] Wang D Wei G Ma J Cheng S Jia L Song X. Identification of the prognostic value of ferroptosis-related gene signature in breast cancer patients. BMC Cancer (2021) 21:645. doi: 10.1186/s12885-021-08341-2 34059009 PMC8165796

[36] Ye CL Du Y Yu X Chen ZY Wang L Zheng YF. STEAP3 affects ferroptosis and progression of renal cell. carcinoma through the p53/xCT pathway. Technol Cancer Res Treat (2022) 21:15330338221078728. doi: 10.1177/15330338221078728 35275508 PMC8921746

[37] Wu HT Chen WJ Xu Y Shen JX Chen WT Liu J. The tumor suppressive roles and prognostic values of STEAP. family members in breast cancer. BioMed Res Int (2020) 2020:9578484. doi: 10.1155/2020/9578484 32802887 PMC7421016

[38] Schmid P Cortes J Pusztai L McArthur H Kümmel S Bergh J. Pembrolizumab for early triple-negative breast cancer. N Engl J Med (2020) 382:810–21. doi: 10.1056/NEJMoa1910549 32101663

[39] Schmid P Rugo HS Adams S Schneeweiss A Barrios CH Iwata H. Atezolizumab plus nab-paclitaxel as first-line treatment for unresectable, locally advanced or metastatic triple-negative breast cancer (IMpassion130): Updated efficacy results from a randomised, double-blind, placebo-controlled, phase 3 trial. Lancet Oncol (2020) 21:44–59. doi: 10.1016/S1470-2045(19)30689-8 31786121

[40] Mito I Takahashi H Kawabata-Iwakawa R Ida S Tada H Chikamatsu K. Comprehensive analysis of immune cell enrichment in the tumor microenvironment of head and neck squamous cell carcinoma. Sci Rep (2021) 11:1–9. doi: 10.1038/s41598-021-95718-9 34373557 PMC8352955

[41] Shi ZZ Tao H Fan ZW Song SJ Bai J. Prognostic and immunological role of key genes of ferroptosis in pan-cancer. Front Cell Dev Biol (2021) 9:748925. doi: 10.3389/fcell.2021.748925 34722530 PMC8548644

[42] Song X Wang X Liu Z Yu Z. Role of GPX4-mediated ferroptosis in the sensitivity of triple. negative breast cancer cells to gefitinib. Front Oncol (2020) 10:597434. doi: 10.3389/fonc.2020.597434 33425751 PMC7785974

[43] You KS Yi YW Cho J Seong YS. Dual inhibition of AKT and MEK pathways potentiates the. anti-cancer effect of gefitinib in triple-negative breast cancer cells. Cancers (2021) 13:1205. doi: 10.3390/cancers13061205 33801977 PMC8000364

