# Supplementary material for: Upregulation of the ferroptosis-related STEAP3 gene is a specific predictor of poor triple-negative breast cancer patient outcomes
Source: Front Oncol. 2023 Mar 31;13:1032364. doi: 10.3389/fonc.2023.1032364 (PMC10102497; doi:10.3389/fonc.2023.1032364)
Supplement: Supplementary file 1 [file DataSheet_1.docx]

**Supplementary Figure**

**Figure S1.** *STEAP3* expression in non-TNBC cell lines. No significant differences in *STEAP3* expression were observed in MCF-7, T-47D, or BT-474 cells compared with normal MCF-10A breast cells (P>0.05).


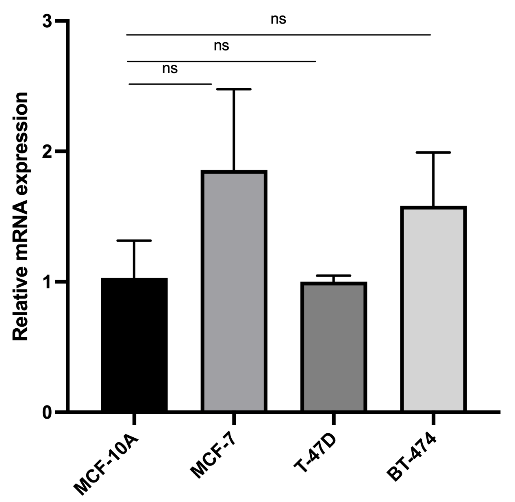


**Figure S2.** *STEAP3* expression in non-TNBC cell lines. No significant differences in *STEAP3* expression were observed in MCF-7 or BT-474 cells compared with normal MCF-10A breast cells (P>0.05).


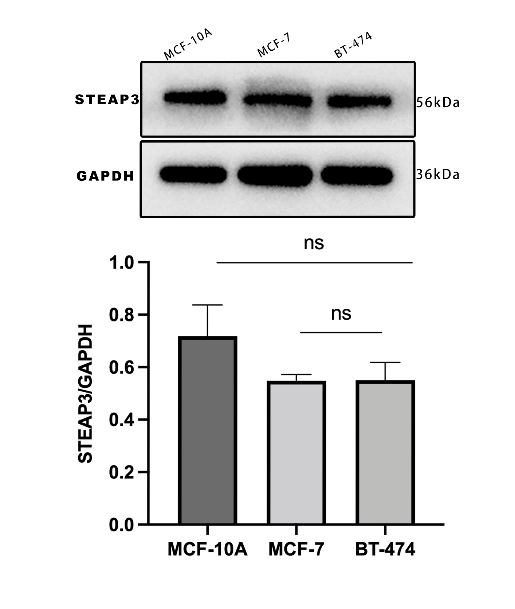


**Figure S3.** In all molecular subtypes of breast cancer, the expression of *STEAP3* was not related to OS based on Kaplan Meier plotter data analyses.
